# A clinical case study of a psychoanalytic psychotherapy monitored with functional neuroimaging

**DOI:** 10.3389/fnhum.2013.00677

**Published:** 2013-10-23

**Authors:** Anna Buchheim, Karin Labek, Steffen Walter, Roberto Viviani

**Affiliations:** ^1^Institute of Psychology, University of InnsbruckInnsbruck, Austria; ^2^Department of Psychosomatic Medicine and Psychotherapy, University of UlmUlm, Germany; ^3^Department of Psychiatry and Psychotherapy III, University of UlmUlm, Germany

**Keywords:** psychoanalysis, fMRI, psychotherapy process Q-Set, single case studies, attachment

## Abstract

This case study describes 1 year of the psychoanalytic psychotherapy using clinical data, a standardized instrument of the psychotherapeutic process (Psychotherapy process Q-Set, PQS), and functional neuroimaging (fMRI). A female dysthymic patient with narcissistic traits was assessed at monthly intervals (12 sessions). In the fMRI scans, which took place immediately after therapy hours, the patient looked at pictures of attachment-relevant scenes (from the Adult Attachment Projective Picture System, AAP) divided into two groups: those accompanied by a neutral description, and those accompanied by a description tailored to core conflicts of the patient as assessed in the AAP. Clinically, this patient presented defense mechanisms that influenced the relationship with the therapist and that was characterized by fluctuations of mood that lasted whole days, following a pattern that remained stable during the year of the study. The two modes of functioning associated with the mood shifts strongly affected the interaction with the therapist, whose quality varied accordingly (“easy” and “difficult” hours). The PQS analysis showed the association of “easy” hours with the topic of the involvement in significant relationships and of “difficult hours” with self-distancing, a defensive maneuver common in narcissistic personality structures. In the fMRI data, the modes of functioning visible in the therapy hours were significantly associated with modulation of the signal elicited by personalized attachment-related scenes in the posterior cingulate (*p* = 0.017 cluster-level, whole-volume corrected). This region has been associated in previous studies to self-distancing from negatively valenced pictures presented during the scan. The present study may provide evidence of the possible involvement of this brain area in spontaneously enacted self-distancing defensive strategies, which may be of relevance in resistant reactions in the course of a psychoanalytic psychotherapy.

## INTRODUCTION

The empirical investigation of the psychoanalytic process and outcome is of great importance to advance our knowledge of the psychoanalytic theory of treatment. Several studies have demonstrated the efficacy of long-term and short-term psychoanalytic treatment in randomized controlled trials (e.g., Gabbard et al., 2002; Leichsenring et al., 2004; Leichsenring and Rabung, 2008, 2011). Nevertheless, many clinicians and researchers argue that detailed single case studies, a time-honored instrument of psychoanalytic inquiry and knowledge dissemination (Donnellan, 1978; Edelson, 1985; Kächele et al., 2009) are still an essential complement to clinical trials in furthering our understanding of the psychoanalytic process and its relation to outcome (e.g., Kächele et al., 2006, 2009). Single case research has been often indicated as one of the most suitable approach for evaluating psychoanalytic treatments (Wallerstein and Sampson, 1971; Edelson, 1988; Hilliard, 1993). Recently, single case studies based on operationalized instruments have been developed in different domains (e.g., Kazdin, 1982). These efforts have produced psychotherapy studies focusing on computerized text-analytic measures (e.g., Mergenthaler and Kächele, 1996), process and outcome research (e.g., Hilliard, 1993; Orlinsky et al., 2004; Gullestad and Wilberg, 2011), and the combination of psychotherapy research and fMRI (Schiepek et al., 2009, 2013).

The aim of the present study was exploring for the first time the feasibility of single case research of an ongoing psychoanalysis in a neurobiological context using repeated fMRI measurements. We pursued the integration of clinical presentation, of operationalized formal instruments to describe the individual psychotherapeutic process, and of neuroimaging techniques to monitor the psychotherapeutic process on both the clinical and the neural levels. To this end, we collected functional neuroimaging data at monthly intervals from a patient undergoing psychoanalytic psychotherapy during exposure to attachment-relevant pictures (Buchheim et al., 2006, 2008). The main question we wanted to address was the extent to which the data from functional neuroimaging could be brought to bear on our theoretical understanding of the psychoanalytic process. Likewise, we were interested in verifying if existing interpretations of cortical activity gained in controlled experimental settings from neuroimaging studies would maintain their explanatory power in the context of the single case study of a psychoanalytic process. A crucial issue was therefore the existence of an association between symptoms, the character of the relationship with the therapist in individual therapy hours, and variation in the signal from the attachment-relevant scenes probe in the scanner.

## MATERIALS AND METHODS

One year of psychoanalytic therapy of a patient with a chronic depressive disorder and narcissistic traits was assessed at monthly intervals (*N* = 12 sessions) with an established measure for the characterization of therapy (The Psychotherapy Process Q-Set, PQS; Jones, 2000), and with a functional neuroimaging probe that was successfully used to elicit signal in an adult attachment context in a previous study of the psychoanalytic treatment of recurrent depression (Buchheim et al., 2012).

The patient, a 42-years-old female lawyer, suffered from rapidly fluctuating affective states. Waking up the morning she knew that “this will be an easy day” or “this will be a difficult day.” Her capacity for successful work and concentration was reduced when she felt depressed and in a “difficult day mood.” During these occasions she isolated herself, tended to withdraw from relationships, and worked hard to hide her emotional vulnerability. This chronic and fluctuating depressive pathology and a fragile, vulnerable perception of self and others brought her in psychoanalytic treatment.

In order to obtain an objective assessment of the psychotherapy process describing the psychodynamic pattern of the patient and the interaction between the patient and the therapist, one session every 4 weeks (first session of the week) at regular intervals (compatible with interruptions due to vacations and illnesses) was audiotaped, transcribed, and analyzed with the PQS approach (12 sessions in all). The PQS (Jones, 2000; German version: Albani et al., 2007) is a rating instrument designed to provide a basic vocabulary for the description and classification of psychotherapy processes in a form suitable to quantitative analysis (Q-sort methodology). The PQS captures a wide range of events in the psychotherapeutic session attributable to both the therapist’s activity and the patient, including transference manifestations, resistance, and the accompanying affective states.

Functional neuroimaging scans were taken on the same days as the recorded therapy hours. As in a previous study (Buchheim et al., 2012), attachment-relevant scenes were used to capture individual attachment-related features relevant for the psychotherapeutic relationship. In the scanner, the patient looked at the scenes used in a formal measure for the assessment of adult attachment representations (Adult Attachment Projective Picture System, AAP; Buchheim et al., 2006, 2008; George and West, 2012). These scenes were alternately accompanied by sentences neutrally describing their content, or by sentences that referred to the personally relevant content evoked by them as extracted by a previous AAP interview. The contrast of interest was the difference between the signal evoked by the personalized and the neutral textual descriptions of the scenes. This contrast detected neural substrates activated by the appraisal of the personal element in the attachment scenes, at the net of generic activations due to the perceptual encoding of the scenes and reading the textual description.

Note that we did not have access to changes in brain function during therapy, since the functional neuroimaging sessions necessarily took place after, and not during, therapy hours. However, we were aided in our attempt in establishing a link between mind states, therapy, and neural substrates by the oscillations of the patient between “difficult” and “easy” days, a change in mood that may have been relatively stable from the therapy hour to the functional neuroimaging session. Hence, the data we present document changes in these modes of emotional functioning that had consequences on the quality of the therapy hours, rather than the therapy hours themselves. The question of interest was the extent to which clinical data from the therapy hours and neural activation were reciprocally informative of the mental states at the base of the “easy” and “difficult” moods. This focus also allowed us to sidestep the issue of change over the course of therapy, which would possibly be confounded by habituation or learning effects in the neuroimaging data.

### PARTICIPANTS

The *analyst* was a very experienced training analyst with an interest in research. She agreed to take part in our study and to audiotape one therapy session a month for the PQS analysis. She works in a private practice as a psychiatrist.

The *patient* also agreed to take part in the study. She was given information about the study and signed a declaration of her willingness to participate for 1 year and to be assessed with several questionnaires and the functional neuroimaging scans. The treatment was paid by the health insurance. This study was approved by the ethical committee by the University of Ulm in the context of the Hanse-Neuro-Psychoanalysis Study (Buchheim et al., 2008, 2012). The patient gave written informed consent to the publication of the data. However, the case report should be written taking into consideration the need to protect the identity of the patient.

### TREATMENT

This patient was treated with a standard long-term psychoanalysis with a frequency of two face-to-face sessions per week. Standard key techniques included exploration, clarification, and interpretation. Interpretive interventions aimed to enhance the patient’s insight into her repetitive conflicts sustaining her problems; supportive interventions aimed to strengthen abilities that were temporarily inaccessible to the patient’s owing to acute stress (e.g., traumatic events) or were not sufficiently developed (e.g., Fonagy and Kächele, 2009; Shedler, 2010). The establishment of a helping (or therapeutic) alliance is regarded as an important component of supportive interventions. Transference, defined as the repetition of past experiences in present interpersonal relations, constitutes another important dimension of the therapeutic relationship. In psychodynamic psychotherapy, transference is regarded as a primary source of understanding and therapeutic change (e.g., Fonagy and Kächele, 2009). In this low frequency therapy, the analyst followed an intersubjective approach, characterized by the focus on the inner and outer reality of the patient’s self and object representations and the aim to increase the patient’s capacity to differentiate between reality and fantasy by enhancing self-reflection (e.g., Ogden, 1977, 1989; Fonagy et al., 2004; Dreyer and Schmidt, 2008).

### CLINICAL AND BEHAVIORAL OUTCOME VARIABLES

The clinical and behavioral outcome data served different purposes. First, the monitoring of symptoms with self-rating scales documented changes in affective symptoms at the days of the data collection. In clinical studies, these data describe the level of symptoms and document changes during therapy (in the present case study, these measures indicated a substantially stable state over the year of the study, as detailed below). Second, the clinical rating of the hour by the therapist and the PQS documented the exchange between patient and analyst during therapy through the clinical impression and an operationalized assessment instrument. Variation in these data provided correlates to explore with the neuroimaging probe. Third, the AAP interview provided material on core attachment issues specific to the patient for the preparation of the stimuli used in the neuroimaging sessions. Almost as a side product, it also provided an assessment of the attachment pattern of this patient at the beginning of study. The AAP interview, however, is not administrable on a monthly basis and for this reason could not be used as a clinical correlate of the neuroimaging data in the present setting. Fourth, a post-scan self-rating questionnaire was administered to evaluate reported involvement with the stimuli presented during the scan session. These data were meant as an aid in interpreting the fMRI analysis. Finally, the results section also reports on the patient using a more customary clinical description informed by psychoanalytic views. We considered the clinical description an integral part of the results, this being a single case study. This description is meant to provide guidance on the psychopathology of this patient, to be compared with the functional role of neural structures identified in the neuroimaging study.

#### Clinical rating of the hour by the therapist

The analyst rated on a clinical level dichotomously if the 12 sessions were “difficult” or “easy.” According to her documentation the classification in “difficult” or “easy” was very clearly identifiable. The “difficult” sessions started with silence and remained quiet and inhibited. The “easy” sessions started fluently and remained talkative.

#### Psychopathology monitoring with self-rating scales

At each scanning session, the patient filled a number of self-rating scales documenting her psychopathological state. State depressiveness was rated with the Collegium Internationale Psychiatriae Scalarum (CIPS)-depressiveness scale (Zerssen, 1976). This is a self-rating depressiveness scales provided in two parallel series of questions, which may be used in alternative turns in sequential assessments. The general burden of symptoms was gaged with the outcome questionnaire (OQ)-burden subscale (German version: Haug et al., 2004).

#### Psychotherapy process Q-set

The PQS is an operationalized instrument for the characterization of therapy hours (Jones, 2000). It consists of 100 items covering a wide range of aspects in the behavior of the patient and her interaction with the therapist. Unlike most rating instruments, the items are not arranged in predefined groups that considered together provide scores on clinical dimensions identified *a priori*. Instead, a typical use of this instrument in psychotherapy research is the identification of hallmark of hours with specific characteristics. For example, one may attempt to identify items correlating with a negative therapeutic reaction, ascertained clinically in a carefully monitored therapy sample. Among their uses, these items can identify both the unity and coherence of treatment sessions, and detects changes between hours and patients. The PQS-instrument shows excellent inter-rater reliability, item reliability, concurrent, and predictive validity for several studies and various types of treatment samples (see Levy et al., 2012). The inter-rater reliability, assessed for all 100 items and tested by correlating the Q-sorts of multiple raters, is high as evidenced by levels of inter-rater agreement/reliability (kappa ranges from 0.83 to 0.89). Reliability varies from adequate to excellent for individual items, giving values between 0.50 and 0.95 (see Levy et al., 2012). In this study verbatim transcribed sessions were coded by two independent raters, who were blind to all therapy hours. Two independent trained judges rated all 12 psychotherapy sessions and achieved a correspondence of kappa between 0.80 and 0.97.

#### Statistical analysis of behavioral data

Because of the inherently correlational and explorative character of data obtained with the PQS, we investigated the tendency of PQS scores to covary across items with a principal component analysis. To compute significance levels of principal components, we carried out 2000 Monte Carlo simulations in which principal component analyses were computed on data with the same item range and distribution, but varying independently from each other. Significance values were computed as quantiles of the first and second components of the simulations (to test the significance of the first and second component, respectively). Significant components provide evidence that a set of therapy characteristics occur together, suggesting the existence of recurrent interaction dynamics.

Hypothesis testing on PQS items were conducted on the linear trend (the months of therapy from 1 to 12) and on the classification of “easy” and “difficult” hours provided by the analyst. The first test documented the existence of a change in the tendency of these interaction dynamics to occur with different frequencies at the beginning and at the end of the period of the study. The second test constituted an objective verification of the clinical impression of the analyst. Tests were carried out independently on each PQS item, correcting for the multiple comparison using a permutation method with 2000 steps (Blair et al., 1994). In this approach, at each permutation the maximal (minimal) *t*-value obtained from conducting the test on the PQS item was recorded. The significance levels of high (low) *t*-values, with adjustment for multiple testing, were given by the quantiles of the recorded maximal (minimal) *t*-values.

#### Self-rating questionnaire after fMRI sessions

To monitor the extent of emotional involvement and autobiographical character of the three core sentences during the course of the psychotherapy, we administered a self-rating questionnaire to the patient after each fMRI session. In the questionnaire the patient was asked to rate the personalized sentences from the AAP scenes used in the scanner by answering the following two questions: “How much of the sentence applies to you autobiographically?” and “How strong did this sentence move you emotionally?” The patient had to assign a score between 1 and 7, where 1 meant not at all, 4 meant middle intensity, and 7 meant very much.

#### AAP interview

Attachment classification and fMRI-stimuli were derived from the AAP (George and West, 2012), an established and validated interview to assess attachment representations, based on a set of eight picture stimuli. The stimuli are line drawings of a neutral scene and seven attachment scenes (e.g., illness, separation, solitude, death, and threat). The AAP classification system designates the four main adult attachment groups identified using the AAI classification system (secure, dismissing, preoccupied, unresolved). Classifications are based on the rating of several scales (e.g., agency of self, connectedness, synchrony, deactivation) on the basis of verbatim transcripts of the stories to the seven attachment activating stimuli.

Administration involves asking participants in a semi-structured format to describe the scene in the picture, including what characters are thinking or feeling, and what they think might happen next. Three core sentences that represented the attachment pattern of the participants were extracted from the audiotaped responses to each AAP picture stimulus by two independent certified judges (e.g., “A girl is incarcerated in that big room,” “My mother suffered until the end and the ambulance came often”). These sentences were paired to the respective picture to constitute the “personally relevant” trials tailored to each participant. These same pictures, paired to sentences describing only the environment of the depicted situation (e.g., “There is a window with curtains on the left and right,” “There is a bed with a big blanket”) constituted the “neutral” trials (see also Buchheim et al., 2012).

### NEUROIMAGING OUTCOME VARIABLES

The neuroimaging session took place on the same day as the recorded psychotherapy hour. It consisted of the task in the scanner and in the administration of a rating instrument to assess the patient’s reaction to the items presented in the scanner.

#### Neuroimaging task

**FIGURE 1:**
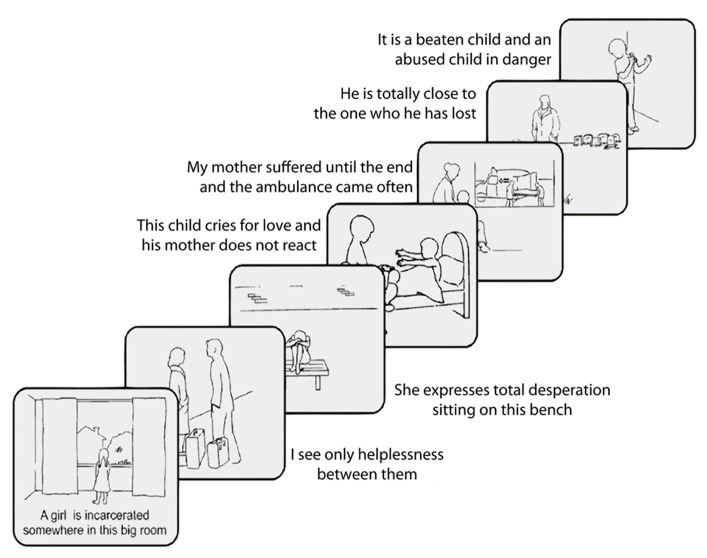
**Schematic representation of the AAP scenes and sentences used in the functional neuroimaging study (example of a personalized trial)**.

In each trial, the patient looked at pictures of attachment-relevant AAP scenes, accompanied by a short descriptive text. Each picture was presented for 20 s, followed by a fixation point for about the same duration (**Figure 1**). The AAP consists of a set of seven of such pictures; this set was repeated 12 times, for a total of 84 trials. Repetitions of the set were divided into two groups: those in which the descriptive text was a neutral rendering of the figures appearing in the scene (*neutral trials*), and those where the description was tailored to core conflicts of the patient as assessed in the initial AAP interview (*personally relevant trials*).

#### Image acquisition

MRI data were recorded using a 3-T Magnetom Allegra head scanner (Siemens, Erlangen, Germany), equipped with a standard head coil. In each session, 508 EPI T_2_*-weighted whole brain volumes were acquired (TR/TE = 2500/30 ms, flip angle 90°, FOV 192 mm, matrix 64 × 64, voxel size 3 mm × 3 mm, slice thickness 3 mm, 44 slices, standard AC–PC orientation). Sessions were repeated in monthly intervals for a year, for a total of 12 sessions.

#### Preprocessing and statistical analysis of neuroimaging data

Data were analyzed with the Statistical Parametric Mapping (SPM) package (Frackowiak et al., 2003), using a voxelwise approach. After realignment and normalization into Montreal Neurological Institute (MNI) space, volumes were smoothed with a Gaussian isotropic kernel (8 mm full width-half maximum). The blood oxygenation level-dependent (BOLD) response function was modeled by convolving the trial onsets with a standard hemodynamic response function. Effects of interest were estimated for each session separately (in a model that included presentation of the scene + textual description combination and whether the combination was personalized or not) and brought to the second level to account for a random effect of sessions (Penny and Holmes, 2007). At the second level, main effects were tested with one-sample *t*-tests. The interaction between quality of the hour and personalized effect was given by an additional second-level regressor indicating whether the hour was “easy” or “difficult.” This regressor is orthogonal to the one-sample *t*-test of the personalized effect (Viviani, 2010).

The main effect of interest of the study was given by the contrast personally relevant vs. neutral, and its interaction with the index of the quality of the session, as indicated by the therapist and its operational characterization through the PQS scores. To identify regions associated with the presentation of the personalized trials, we performed a whole-brain estimation of the model voxel by voxel. The significance levels reported in the text of section “Results” are corrected at cluster level (Poline and Mazoyer, 1993; Friston et al., 1994) for the whole volume.

The post-scan self-rating scales were analyzed separately from neuroimaging data using the freely available package R (The R Foundation for Statistical Computing, www.r-project.org, Vienna, Austria; repeated measures regression: function *lmer*, package lme4, version 2.13.1; Bates and Maechler, 2009). The dependent variable (emotional involvement or extent of autobiographical character of the scene–sentence couple) was modeled in a repeated measurements linear model as an effect of the hour character (“easy” or “difficult”) and the personally relevant AAP scene as fixed effects, and the session and the sentences as grouping variables for the random effects.

## RESULTS

### CLINICAL DESCRIPTION OF THE PATIENT

The patient, a 42-years-old lawyer, suffered since the birth of her first daughter from rapidly fluctuating affective states. From a clinical point of view, the patients had a moderate functioning level. During the so-called “difficult day”-states she isolated herself and tended to withdraw herself in relationships and hide her emotional vulnerability in contrast to the so-called “easy days”-states, where the patient felt self-conscious and full of personal strength. Regarding her personality structure she showed some narcissistic features (Kernberg, 1984; Cain et al., 2008; Pincus and Lukowitsky, 2010), being self-centered and rather achievement oriented. She defined herself frequently via money, success, and reputation. When she felt in her job that clients were not as satisfied with her work as she expected from herself she broke down and was ruminating anxiously if they will come back. This pattern demonstrated that her self-esteem fluctuated according to the gratifying or frustrating experiences in relationships and how she evaluated the distance between the goals and aspirations. Because of her harsh super-ego demand for perfection she was in an instable inner state and self-esteem could be diminished rapidly.

The patient lived in a long-lasting relationship. However, she characterized the relationship with her husband as competitive with respect to their tendency to experience rivalry and envy. Moreover, there was a clear discrepancy between her self-perception and the perception that significant others had of the patient. Although easier days were subjectively felt more pleasant by the patient, her husband reportedly found it very difficult to deal with her. This often led to constant, seemingly unsolvable conflicts and to repeatedly considering separation.

One of her major unconscious defensive structure seemed to circle around fantasies of success and grandiosity, leading to her dependency to be admired by others and to bouts of insecurity disrupting her sense of grandiosity or specialness (for a description of the related dynamic, see Kernberg and Yeomans, 2013).

According to the observations of the analyst collected over 1 year of clinical work, the following topics may be considered key to the psychodynamic understanding of the patient and her treatment:

1. On “difficult” days the patient showed a severely inhibited capacity to think and to express feelings and thoughts and fell into silence. On “easy” days the patient talked expansively and her personality appeared strong.

2. The association of the fluctuating symptoms with unresolved loss experiences and fear due to uncontrollable guilt-feelings.

As we shall see later, these two core issues could be retrieved in the formal assessment of the interaction between the patient and the therapist using the PQS methodology.

From a psychodynamic and biographic perspective the analyst suggested that two events of death were useful to understand the nature of the patient’s symptoms. These events revealed the underlying vulnerability of the patient with respect to this issue and the related latent feelings of helplessness and impotence. When the patient was 30-years old her mother died unexpectedly. She felt guilty, because she was unable to call the emergency doctor in time. Moreover, the tragic loss through death of a colleague some years previously coincided with the birth of her first child, a son. Again the patient felt guilty, because she was not able to reach her colleague in time to be able to help her. Her fluctuating depressive symptoms might be interpreted as the outcome of this defensive structure. On “easy days” her functioning was predominantly characterized by externalization with an increase of activity and personal strength, while on “difficult days” internalization led to inhibition of activity and severe self-doubts. These latter phases were characterized by affective distance between the patient and her object world in an effort to preserve the illusion of control relative to object loss (Modell, 1975).

Since the patient demonstrated a complex chronic affective disorder with difficult personality traits and a rigid defensive structure, there was an indication for long-term psychoanalytic treatment with two sessions per week (Leichsenring and Rabung, 2011). The treatment setting was face-to-face, thus creating a positive stable counterpole to her mood changes. The positive stabilizing effect of the therapy was noticeable early in the treatment even though the total process was taking a very long time. The treatment centered on the deeper understanding of her uncontrollable mood-shifts and her impaired self-perception and perception of others. The question of failure and/or the continuing of the analytic work were constantly present. The transference relationship was mirrored by her experiences of loss: she failed to prevent the unexpected deaths, and for a long time the analyst and the patient failed to prevent the unexpected mood-shifts and to find ways how she could regulate and stabilize her affective instability. Gradually, the patients internalized a better perception of herself and it became easier for the patient to succeed regulating her mood toward the state characterizing “easy” days. One major focus of the treatment was to increase the patient’s ability to react timely in case of severe events like illness or death, and therefore to be able to process these potential traumatic events in a more controlled and integrated way.

### ATTACHMENT DATA

The patient was administered the AAP interview at the beginning of the fMRI experiments and 1 year later. The AAP interview had two purposes. On the one hand we assessed the patient’s attachment representation at the beginning of the fMRI assessment and on the other hand we extracted core sentences of the patient’s narratives in the AAP interview as the personalized stimulus material in the fMRI setting (see Section “Neuroimaging Task”). The patient was classified as unresolved (i.e., disorganized). Unresolved stories typically leave characters without protection, describe feelings of extreme mental distress that have not been diminished or transformed, or leave threatening images looming without addressing them further. The patient demonstrated a lack of resolution especially in the AAP Picture “Cemetery” where the loss of the father was associated with mourning, loneliness and a present dialogue with the dead father, which indicated a spectral quality.

### ANALYSIS OF SELF-RATING SCALES

**FIGURE 2:**
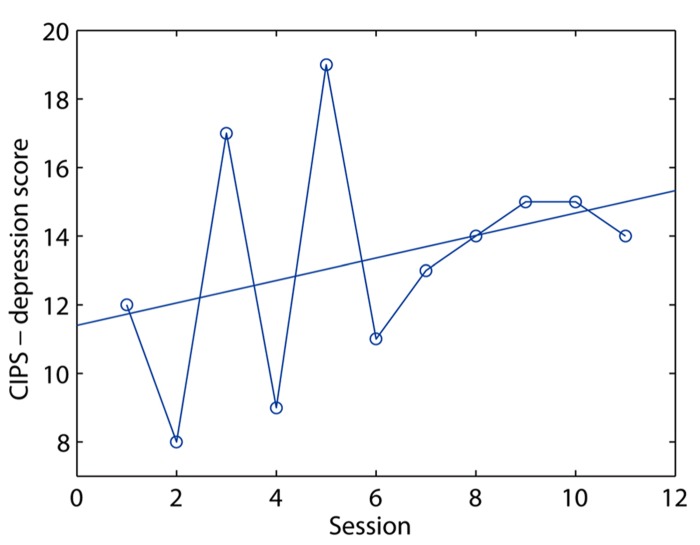
** Plot of depressiveness scores (*y*-axis) over the 12 months of the study (*x*-axis)**.

Analysis of the CIPS-depressiveness score gave a mean value of 12.2 (SD 5.2, range 8–19), indicating affective symptoms of moderate intensity. The regression of the scores over time failed to demonstrate the existence of changes. Nominally, in the examined monthly sessions the patient became more depressed during the year she was monitored (**Figure 2**), but the result was far from significant (*t* = 1.05; df = 10, *p* = 0.34, two-tailed).

The general burden of symptom, as measured by the OQ subscale, was on average 41.75 (SD 5.0, range 33.53), indicating alternating degree of symptom severities crossing the line of norm values (Haug et al., 2004). Like depressiveness, the symptom burden also increased lightly, but not significantly, during this period (*t* = 1.16, df = 10, *p* = 0.27, two-tailed).

### PQS SCORES

The analysis of the PQS scores took place in three steps. In the first step, we undertook an explorative analysis to answer the question of whether there were consistent changes over therapy hours across different items of the PQS, by carrying out a principal component analysis of the PQS scores. This analysis aimed at detecting items that were high or low together in the same hour, without imposing *a priori* constraints on what these items should be, as would be the case if items had been grouped into preformed scores. We also looked at whether these changes were consistent with a linear trend (i.e., a gradual change over time). In the second step, we looked at the existence of items that were associated with the analyst’s classification of the hours in good and bad. In the final step, we looked at whether changes detected during the explorative analysis related to the changes associated with the analyst’s judgment.

In the principal component analysis of PQS scores, the first detected component, which explained about 32% of the variance of PQS items over time, was highly significant (*p* < 0.001). A second component only reached trend significance (*p* = 0.06), explaining 16.7% of the overall variance over time. Further components, explaining 13% of the variance of less, failed to reach significance even at trend level. The 10 items scoring highest in the first and second components are shown in **Table 1**.

**Table 1:** Ten highest scoring items from the principal component analysis of the PQS.

Polarity	Weight	PQS item
**First component**		
+	0.41	25: Patient has difficulty beginning the hour.
+	0.33	12: Silences occur during the hour.
-	-0.30	54: Patient is clear and organized in self-expression.
+	0.25	7: Patient is anxious or tense (vs. calm and relaxed).
-	-0.21	13: Patient is animated or excited.
-	-0.21	23: Dialog has a specific focus.
-	-0.20	74: Humor is used.
+	0.16	8: Patient is concerned or conflicted about his or her dependence on the therapist (vs. comfortable with dependency, or wanting dependency).
-	-0.16	87: Patient is controlling.
+	0.15	15: Patient does not initiate or elaborate topics.
**Second component**		
-	-0.29	30: Discussion centers on cognitive themes, i.e., about ideas or belief systems.
-	-0.28	31: Therapist asks for more information or elaboration.
+	0.27	40: Therapist makes interpretations referring to actual people in the patient’s life.
-	-0.25	45: Therapist adopts supportive stance.
+	0.25	63: Patient’s interpersonal relationships are a major theme.
-	-0.22	12: Silences occur during the hour.
-	-0.21	66: Therapist is directly reassuring
-	-0.20	95: Patient feels helped.
+	0.19	1: Patient verbalizes negative feelings (e.g., criticism, hostility) toward therapist (vs. makes approving or admiring remarks).
+	0.19	49: The patient experiences ambivalent or conflicted feelings about the therapist.

Several items in the principal component analysis scored negative values. The PQS manual contains specific indications to score items as distinctively low. In the first component, a low score on item 54 is given for rambling or incoherent communications, and on item 23 for lack of a guiding discourse thread; on item 13 for the patients appearing bored or dull, and on item 74 grave or somber. Considered together with items with high scores (whose interpretation is immediate), they show that component one prevalently collected items suggesting difficult or inhibited communication of the patient toward the analyst, with frequent phases of silence. These occurred together with other items suggesting the presence of a tense, sober mood (items 7, 13, 74).

The second component appears to characterize form and content of the intervention of the analyst (items 31, 40, 45, 63, 66) and the sometimes difficult reaction of the patient to them (items 1, 49, 95).

**FIGURE 3:**
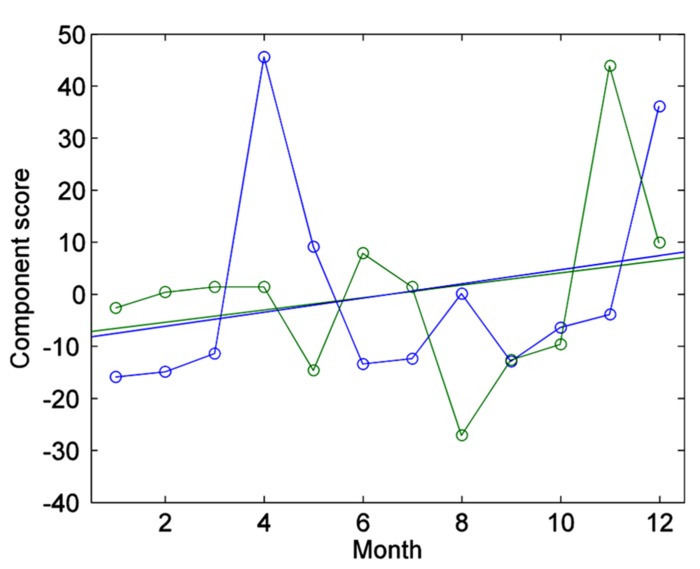
**Plot of first component scores over time, together with the respective linear trends (on the *x*-axis by the monthly session).** In blue, first component scores; in green, second component scores. Both component scores display a small tendency to increase over time, which however was not significant.

We then tested the existence of a linear trend in the changes over time in these component scores. This would have been the case, for example, if the character of the hours changed over the year of therapy, and these components reflected this systematic change. However, the regression of the component scores on the time trend was not significant (first component: *t* = 0.99, df = 9, *p* = 0.35, two-tailed; second component: *t* = 0.82, df = 9, *p* = 0.43, two-tailed), suggesting that they did not change over time (**Figure 3**). Even if the main components did not appear to reflect a change over time, it is conceivable that some other isolated item did. To verify this hypothesis, we tested the regression of each item score over time separately, correcting the significance level for the 100 tests. Also this analysis failed to detect items reflecting a change over the year of therapy. The item that was most associated with time was item 76 (“Therapist suggests that patient accept responsibility for his or her problems,” which however failed to reach significance (*t* = 4.22, *p* = 0.14, two-tailed corrected for multiple comparisons). In summary, change over time in the PQS scores did not document a systematic change after 1 year of therapy relative to the beginning of the monitoring period.

In the second step of the analysis we looked at the existence of items that were associated with the analyst’s classification of the hours in “easy” and “difficult.” Both easy and difficult hours occurred during this year, and a logistic regression of the occurrence of easy hours over time showed the absence of a significant time trend (*z* = -0.53, *p* = 0.60). The separate regression of each PQS item on the analyst indicator of the quality of the hour detected three significant items, after correcting significance levels for multiple comparisons: item 12 (“Silences occur during the hour”), *t* = -9.16, *p* = 0.004 (two-tailed, corrected); item 61 (“Patient feels shy and embarrassed (vs. un-self-conscious and assured.),” *t* = -5.76, *p* = 0.03; item 54 (“Patient is clear and organized in self-expression”), *t* = 5.39, *p* = 0.04. A fourth item reached trend significance, item 7 (“Patient is anxious or tense (vs. calm and relaxed).”), *t* = -4.95, *p* = 0.063.

Finally, we looked at whether changes detected during the explorative principal component analysis in the form of component scores related to the changes associated with the analyst’s judgment. There was a significant association between the first component scores and the analyst’s indicator of the quality of the hour (*t* = -5.03, df = 9, *p* = 0.0006). The second component, in contrast, was not significantly associated (*t* = 1.01, df = 9, *p* = 0.33).

In summary, there was at least one set of PQS items that changed together across therapy hours. These changes were not associated with a time trend, indicating stability of the underlying psychotherapy pattern; however, they were associated with the occurrence of “easy” and “difficult” days. This result did not change if the PQS items were regressed individually on time and day difficulty.

### CORE PSYCHODYNAMIC FEATURES OF THE PATIENT AND PQS RESULTS: AN EXPLORATORY COMPARISON

We compared the clinical features of “difficult” and “easy” days with the first component from the PQS, obtained independently from information on the day difficulty (see **Table 2**). This comparison revealed convergent patterns. The clinical description of the analyst, emphasizing the difficulties of expression of the patient, is consistent with the items in the first component detailing inhibited communication, silence, or ineffective content on difficult days. The identification by the analyst of unresolved feelings of loss corresponds to the items related to tense and sober mood. We conclude that the PQS analysis could validate the subjective evaluations of the analyst.

**Table 2:** Clinical characteristics compared to PQS-items (principal component analysis).

Clinical characteristics	PQS-items
On “difficult” days the patient showed a severely inhibited capacity to think and to express feelings and thoughts and fell in silence.	Item 25: Patient has difficulty beginning the hour.
	Item 12: Silences occur during the hour. 15: Patient does not initiate or elaborate topics.
	Item 15: Patient does not initiate or elaborate topics.
The association of the fluctuating symptoms with unresolved loss experiences and fear due to uncontrollable guilt-feelings	Item 7: Patient is anxious or tense (vs. calm and relaxed).
	Item 8: Patient is concerned or conflicted about his or her dependence on the therapist (vs. comfortable with dependency, or wanting dependency).

### ANALYSIS OF POST-SCAN SELF-RATING QUESTIONNAIRE

The patient was asked after each fMRI session to rate personalized sentences from the fMRI task with respect of self-involvement and autobiographical content (see Section “Materials and Methods”). The analysis of emotional self-involvement revealed that the rating was on the whole significantly higher in the fMRI sessions that followed “easy” therapy hours (*t* = 2.08, df = 9, *p* = 0.03, one-tailed). This result did not change if the autobiographical rating was added as a confounding covariate to the model (*t* = 2.08). This expanded model also revealed that the autobiographical rating was in the individual items associated with the level of emotional involvement rating (*t* = 3.9, df = 193, *p* < 0.001). In contrast, there was no significant change in ratings of the autobiographical character of the personalized sentences in association of the quality of the hour (*t* = 1.27, df = 9, *p* = 0.12 one-tailed).

In summary, these self-rating data confirmed the existence of a qualitative difference between “easy” and “difficult” days that involved the stimuli presented in the scanner through the tendency of a higher self-rated emotional involvement on “easy” days. However, over and above this association, there was an even stronger association at each individual rating between the level of self-involvement and the level of autobiographical character of the scene + text combination.

### NEUROIMAGING RESULTS

**FIGURE 4:**
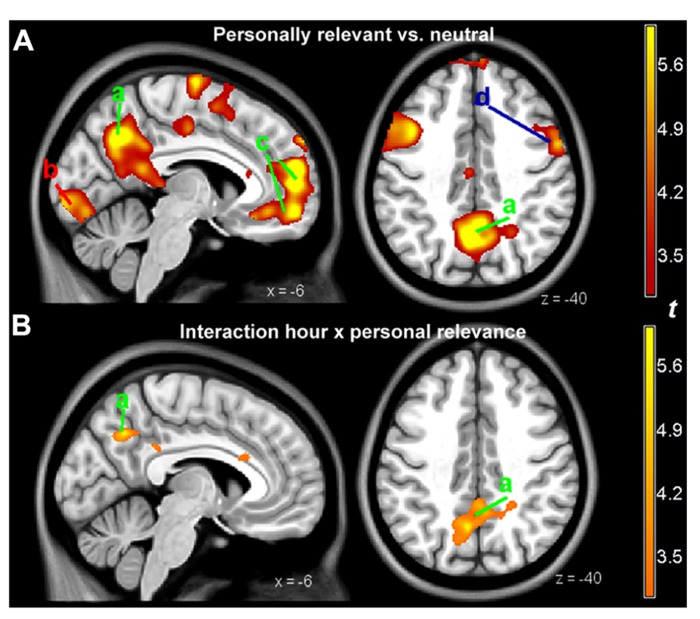
**(A)** Parametric maps of activations detected in the personally relevant vs. neutral contrast, overlaid on a template image. **(B)** Parametric maps of the interaction of the same contrast with hour quality, as rated by the therapist. Slices positioned at MNI coordinates *x* = -6 (left) and *z* = 40 (right). For illustration purposes, the parametric map was thresholded at *p* < 0.005, uncorrected, and a cluster size of 150 voxels (1.2 cm^3^). a, precuneus and posterior cingulate, active at both the contrast personally relevant vs. neutral and its interaction with hour quality; b, calcarine cortex; c, perigenual medial prefrontal cortex; d, dorsolateral prefrontal cortex. Areas a and c (labeled in green) belong to the “default network system”; area d to the dorsal attentional network (in blue). The red label b refers to primary visual areas.

When viewing the pictures described by personalized text, relative to those with neutral descriptions, the patient activated several areas, prevalently on the left. The most prominent activations involved the ventrolateral and the dorsolateral prefrontal cortex, the perigenual portion of the medial prefrontal cortex, the posterior cingulate and precuneus, the middle temporal gyrus, and the anterior tip of the inferior temporal gyrus, and the occipital/calcarine cortex (see **Figure 4A** and **Table 3**). No area was significantly more active when looking at the neutral scenes.

**Table 3:** Activations for the contrast personalized vs. neutral.

MNI coord. (mm)	Brodmann area	peak *t*	*p* (peak lev.)	*k*	*p* (cl. lev.)
-50 30 -2	L Inf. frontal orb. (BA45)	13.84	0.001	10393	<0.001
-46 -2 -46	L Inf. temporal (BA20)	11.67	0.007	s.c.	
-68 -52 8	L Mid. temporal (BA21)	10.67	0.020	s.c.	
-8 56 20	L Med. Sup. frontal (BA10)	8.38	0.148	3131	<0.001
-6 56 8	L Med. Sup. frontal (BA10)	8.03	0.197	s.c.	
-12 36 -8	L Mid. frontal orb. (BA11)	7.01	0.440	s.c.	
22 -100 10	R Sup. occipital (BA17)	7.84	0.230	2787	<0.001
8 -86 0	R calcarine (BA17)	7.43	0.319	s.c.	
20 -88 2	R calcarine (BA18)	6.62	0.575	s.c.	
-4 -10 76	L Suppl. motor area (BA6)	6.48	0.627	834	0.002
-4 2 58	L Suppl. motor area (BA6)	4.58	0.999	s.c.	
-12 8 74	L Sup. frontal (BA6)	4.22	1.000	s.c.	
54 -6 52	R Precentral (BA6)	5.65	0.904	800	0.002
54 4 38	R Precentral (BA6)	5.52	0.932	s.c.	
42 14 30	R Inf. frontal Operc. (BA48)	4.91	0.995	s.c.	
68 -36 -2	R Mid. temporal (BA21)	5.22	0.976	245	0.372
62 -34 10	R Sup. temporal (BA22)	4.63	0.999	s.c.	
66 -42 20	R Sup. temporal (BA22)	4.49	1.000	s.c.	
-4 -14 44	L Mid. cingulum (BA23)	5.19	0.979	199	0.561
-10 -20 50	L Mid. cingulum (BA23)	3.95	1.000	s.c.	
-40 -46 56	L Inf. parietal (BA40)	5.08	0.987	173	0.686

*Shown are peaks at least 8 mm apart from clusters of at least 150 voxels (1.2 cm^3^), and uncorrected significance *p* < 0.005. Explanation of abbreviations: MNI coord. (mm.), Montreal Neurological Institute coordinates (in millimeters); *p* (peak lev.), significance level, peak correction according to random field theory; *k*, cluster size, in 2 × 2 × 2 mm. voxels; *p* (cl. lev.): significance level, cluster correction according to random field theory; s.c., same cluster; L, left; R, right; Inf., inferior; Mid., middle; Med., medial; Sup., superior; Orb., orbital; Suppl., supplementary; Operc., operculum.
*

The interaction of the effect of personal relevance with goodness of therapy hours was significant in the posterior cingulate/precuneal region (MNI coordinates, *x*, *y*, *z*: -6, -60, 40, *t* = 6.7, cluster size in voxels: 633, *p* = 0.017). Here, the signal while looking at personalized scenes was higher when the therapy hour was bad. This area, shown in **Figure 1B**, was part of the medial prefrontal network that was associated with viewing personalized scenes (**Figure 1A**). Other, smaller areas detected in the interaction failed to reach significance. No significant interaction was observed in the opposite direction.

We also tested the interaction between the effect of personal relevance and a linear time trend, to detect changes in activation that developed during the year of therapy. In the interaction with the positive time trend, a cluster extending from the left post-central gyrus to the middle frontal gyrus was significant (MNI coordinates, *x*, *y*, *z*: -54, -12, 40, *t* = 10.2, cluster size in voxels: 1410, *p* < 0.001). This interaction partially overlapped with the prefrontal interaction in **Figure 4A** (d, dorsolateral prefrontal cortex). No effect was observed in the interaction with a negative time trend.

## DISCUSSION

Recently, the issue of the relationship between Freudian thought or psychoanalytic theory and technique more generally and neuroscience has been the object of renewed interest (Carhart-Harris et al., 2008; Carhart-Harris and Friston, 2010; Solms and Panksepp, 2012; Zellner, 2012; Schmeing et al., 2013). In the present study, we attempted to integrate a clinical description of the psychoanalytic process with two empirical instruments, one providing an operationalized assessment of the therapeutic interaction, and the other information on brain activity based on a functional neuroimaging probe. Our aim was to explore the extent to which the two main mental states of the patient and their effect on the psychoanalytic interaction could be observed not only at the clinical level, but also through the data delivered by these two additional instruments.

Analysis of the symptomatic scales gave the picture of a patient with affective symptom severity of moderate intensity, occurring in a patient with a personality with narcissistic features, as described in detail in Section “Results.” The unresolved attachment pattern emerging from the AAP interview is consistent with the analyst’s clinical presentation and with recent attachment data on patients, comorbid with borderline personality disorder and narcissistic personality disorder (Diamond et al., 2012).

The analysis of the PQS data showed that sessions differed along a main axis, defined by the first component. This component was highly correlated with the judgment of the analyst on the quality of the sessions. This analysis revealed that “easy” hours were associated with items describing the deeper understanding of relationship issues, “difficult” hours with silence in the therapy hours and difficulties of the patient to feel at ease. Furthermore, there was no evidence in the PQS data of a linear trend over time that reflected systematic changes from the initial to the final phases of the year monitored by the study. In summary, the main change across sessions present in the PQS data was the one documented by the analyst through her judgment in a phase of therapy where the patient remained stable. This source of change was not associated with a time trend, as “easy” or “difficult” days did not occur more often at the beginning or end of the observation year. This allows excluding the confounds of habituation or learning effects from the regressor representing quality of the hour.

**FIGURE 5:**
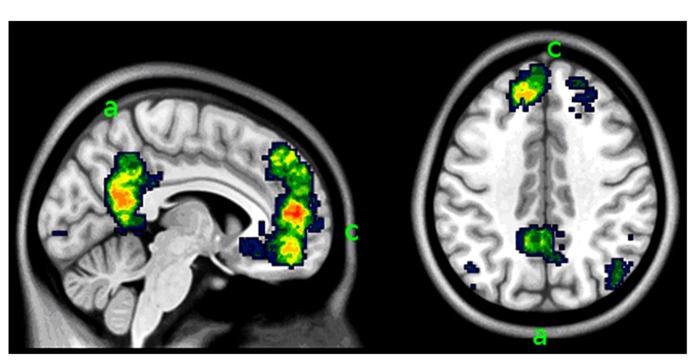
**Areas in the medial face of the brain associated with self-representation.** This image synthesizes data in the neuroimaging literature of studies concerned with self-referentiality using automated keyword search and meta-analytic methods (from www.neurosynth.org, search key “self-referential”; Yarkoni et al., 2011). Slices positioned at MNI coordinates *x* = -6 (left) and *z* = 40 (right). The comparison with the activation detected in the study in the contrast personally relevant vs. neutral contrast (**Figure 4**) shows correspondence of activation in the areas in the medial aspect of the brain: the precuneus and posterior cingulate (a) and the perigenual medial prefrontal cortex (c).

The activation pattern in the contrast of the main effect personally relevant vs. neutral (**Figure 4A**) was characterized by the presence of two main groups of areas. The first included areas that are often active in functional neuroimaging studies and that are known to be active while carrying out a focused task (Duncan and Owen, 2000). This group includes the ventrolateral and the dorsolateral prefrontal cortex, and the occipital/calcarine cortex (for visually presented stimuli). The second group may be considered more specific for the material used in the present study, and included areas in the medial wall (anterior cingulate, and the posterior cingulate and precuneus). The activation pattern of these areas was consistent with the activation found in studies in the literature in which participants were asked to judge the degree to which stimuli presented during the scan were attributed to the self, or were felt to be part on oneself/one’s own description (**Figure 5**; for a systematic review and meta-analysis of the literature, see van der Meer et al., 2010; Qin and Northoff, 2011). The medial prefrontal cortex may also be associated with changes after the therapy of affective disorders (Messina et al., 2013). We therefore considered the areas in this second group as those most likely involved in processing the personally relevant content of the stimuli.

Within this pattern of activation of areas associated to the self and personal relevance, the posterior cingulate cortex was modulated by the interaction with the quality of the therapy hours that had immediately preceded the scan. This association represent evidence of a neural substrates accompanying opposing mental states that, as shown in the self-rating scales, the judgment of the analyst on the quality of the hour, and the formal instrument for assessing the therapeutic exchange, represented a coherent constellation of internally experienced and interpersonally exchanged affect.

The posterior cingulate cortex has been shown in other studies to be modulated by self-distancing from negatively valenced pictures presented during the scan (Koenigsberg et al., 2010) or when down-regulating the reaction to a negative stimulus by self-distraction (Kanske et al., 2011). Of particular interest in the present context is the study by van Reekum et al. (2007), in which gaze fixations were recorded while participants viewed aversive scenes and were left free to choose the down-regulating strategy. This area highly correlated with the amount of eye movements of the participants, who were directing their gaze so as to avoid the focal area of the image where the disturbing content was represented. This area was also reported to be active in regulation strategies adopted by patients with personality disorders characterized by poor emotion regulation (Koenigsberg et al., 2009; Doering et al., 2012; Lang et al., 2012).

The self-rating data collected after the scan confirmed the association between the enactment of a self-distancing strategy from the material and the quality of the hour. On “difficult” days, the patient indicated that her overall emotional involvement with the visuotextual material was lower than on the “easy” days. This corresponded to a higher activity in the posterior cingulate area, associated in the previous studies with self-distancing emotion regulation strategies. In view of the documented association between the quality of the hour and the quality of the interaction with the therapist, and the clinical judgment of the therapist himself, the present study provides evidence on the possible involvement of the posterior cingulate area in spontaneously enacted self-distancing emotion-handling strategies representing defensive maneuvers in the course of a psychoanalytic therapy.

Among the areas active in the contrast personally relevant vs. neutral there were also areas prevalently involved in attentional processes (dorsolateral prefrontal cortex; **Figure 4A** letter c). Also this area was modulated during the year of therapy, showing a progressive increase of the signal due to personally relevant trials. This suggests a dissociation of the areas detected in the contrast personally relevant vs. neutral, with the posterior medial area associating with quality of the hour, and the dorsolateral prefrontal areas associating with change over time. The change over time in the dorsolateral prefrontal cortex might be due to a progressive loss of attentional pull of the non-relevant trials, or to the increased recruitment of attentional resources in looking at scenes in the personally relevant trials. From a clinical point of view it could mean that the patient was more effective in appraising and reflecting on her own personal core attachment-related issues.

There are several noteworthy limitations of this study. First, treatment did not follow a manualized psychoanalytic psychotherapy. However, it was conducted by adhering to specific core techniques, as described in section “Materials and Methods,” by a very experienced psychoanalyst. Second, in the attachment paradigm used in the scanner no pictures without attachment content were present. This is consistent with the choice to investigate personal relevance in the context of material likely to evoke core emotional issues, as in previous work (Buchheim et al., 2012). Future work will have to address the issue of the neural response to attachment pictures of the kind used in the AAP in comparison with neutral pictures of similar content and complexity, but differing attachment relevance and interpersonal quality or emotionality, and its capacity to capture affective psychopathology. Third, the fluctuation between two cognitive–emotional states (easy and difficult days, easy and difficult sessions) may have been indicative of pattern transitions that may be analyzed with approaches focusing on self-organization and non-linear dynamics in psychotherapy (see e.g., Boston Change Process Study Group, 2005; Schiepek et al., 2009, 2013). However, this aspect of the psychotherapeutic interaction fell outside of the scope of the present study.

In summary, this case report gives indications on the interplay between activity in neural circuits and quality of the psychotherapeutic sessions in the context of psychoanalytic process research. In this specific single case, major characteristics of the patient’s defensive structure could be demonstrated on a behavioral and neural level and validated the subjective evaluation of the analyst. Specifically, affective distancing has been identified in the literature as a hallmark defensive maneuver in personality organization with narcissistic traits (Modell, 1975). Using functional neuroimaging, we were able to objectify the defensive structure of this patient during this phase of psychoanalytic treatment and the occurrence of difficult sessions.

## Conflict of Interest Statement

The authors declare that the research was conducted in the absence of any commercial or financial relationships that could be construed as a potential conflict of interest.
